# RNA Interference in Plant Interactions with Pathogenic Microorganisms: A Weapon or a Liability?

**DOI:** 10.3390/cimb48010021

**Published:** 2025-12-25

**Authors:** Artemii Ivanov, Tatiana Golubeva

**Affiliations:** 1 Institute of Cytology and Genetics SB RAS, Novosibirsk 630090, Russia; a.ivanov2@g.nsu.ru; 2 Department of Natural Science, Novosibirsk State University, Novosibirsk 630090, Russia; 3 Higher School of Living Systems, Institute of Medicine and Life Sciences, Immanuel Kant Baltic Federal University, Kaliningrad 236041, Russia

**Keywords:** cross-kingdom RNAi, host-induced gene silencing (HIGS), spray-induced gene silencing (SIGS), plant immunity, pathogens, plant protection

## Abstract

The RNA interference machinery is crucial for regulating the activity of both native and foreign genes across all eukaryotes. The core protein families involved in this process are Dicer-like, Argonaute, and RNA-dependent RNA polymerase. However, plants exhibit remarkable diversity within each family and extensively use RNA interference mechanisms in their intricate immune responses. This review examines the role of RNA interference in plant interactions with various pathogens, including viruses, viroids, fungi, oomycetes, and bacteria. Plant diseases cause an estimated $220 billion in annual damage, with microorganisms accounting for approximately $150 billion. Hence, the focus is on the most severe plant diseases, specifically those caused by fungi and viruses. Additionally, recent biotechnological advancements are discussed, with an emphasis on the application of RNA interference for the development of novel plant defence strategies.

## 1. Introduction

**Figure 1:**
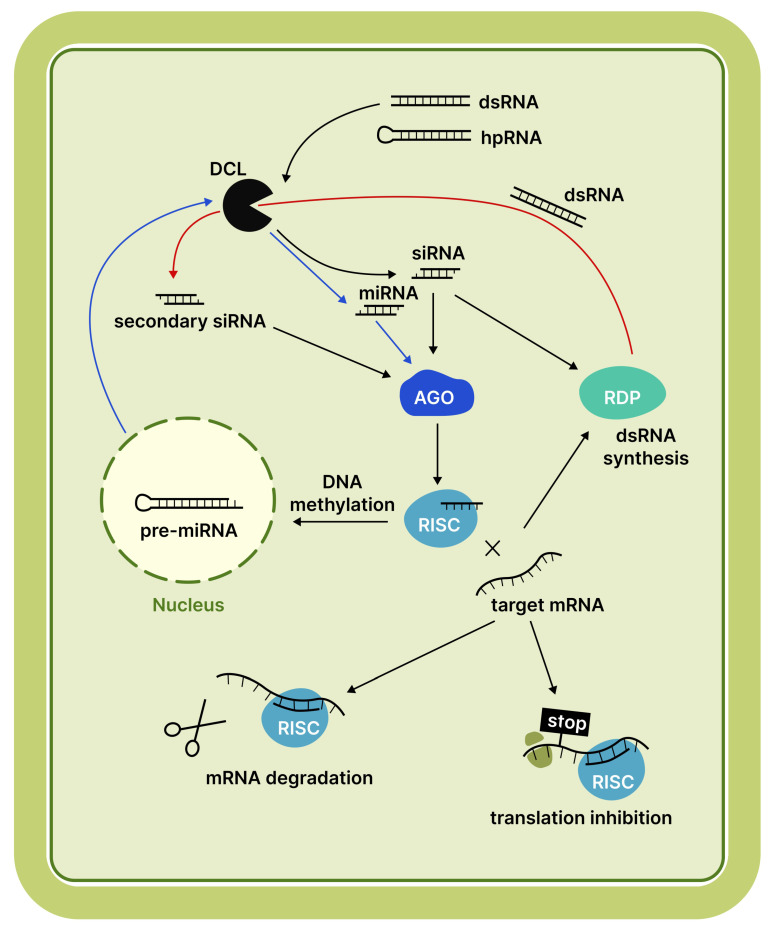
Schematic representation of the RNAi mechanism in plants. Blue arrows indicate the miRNA pathway, while red arrows indicate the secondary siRNA pathway. The specific RNAi outcome depends on the RNA substrate (pre-miRNA or dsRNA), its origin, the specific DCL protein responsible for processing, and the AGO protein that binds the resulting sRNA. Target mRNA slicing by the RISC is required to prime secondary siRNA production [4]. Abbreviations: DCL, Dicer-like protein; AGO, Argonaute protein; RDR, RNA-dependent RNA polymerase; RISC, RNA-induced silencing complex.

RNA interference (RNAi), one of the oldest antiviral defence mechanisms found in all eukaryotes, regulates gene expression and suppresses the activity of mobile elements [1]. RNAi is activated when a protein from the Dicer or Dicer-like (DCL) family, possessing RNase III activity, detects double-stranded RNA (dsRNA) regions, such as hairpins [2]. These proteins cleave dsRNA into short duplexes, typically 18–21, 22, or 24 nucleotides in length. These short duplexes are referred to as small RNAs (sRNAs) [3]. Subsequently, one strand of the duplex loads into an Argonaute (AGO) protein to form the RNA-induced silencing complex (RISC). This complex facilitates post-transcriptional gene silencing (PTGS) by cleaving target messenger RNA (mRNA) or suppressing translation. Alternatively, transcriptional gene silencing (TGS) can be observed when RISC triggers the methylation of complementary DNA [4]. A schematic representation of the plant RNAi mechanism is provided in Figure 1.

In plants, small RNA movement is not confined to the cellular level [5]. They can traverse neighbouring cells through plasmodesmata. This process has been shown to be regulated by microtubules, preventing the binding of AGO proteins to sRNAs [6]. Of even greater significance is their systemic translocation via phloem [7,8,9], enabling the dissemination of the locally generated signal. The mechanism of this long-distance migration appears to involve, but is not limited to [10,11], their encapsulation in extracellular vesicles with RNA-binding proteins, such as AGO [12].

Why have plants evolved such a diverse array of sRNA-interacting proteins and trafficking mechanisms? Initially, it was assumed that these components primarily functioned within the immune system to neutralise viruses and mobile genetic elements [13,14]. In addition, it has been known for quite a long time that dsRNAs synthesised in plants are transferred to insects and their larvae in the process of feeding [15,16]. A groundbreaking discovery by Weiberg et al. [17] demonstrated the exchange of interfering RNAs between a plant and a pathogenic fungus. They found that *Botrytis cinerea* employs small interfering RNA (siRNA) during infection. Notably, mutant strains lacking siRNA production exhibited decreased virulence. Over the following decade, researchers documented the transfer of siRNAs into plants from fungi [18], oomycetes [19], and a parasitic plant [20]. Furthermore, RNA transfer occurs between plants and viroids: sRNAs generated after DCL processing of viroids can suppress host gene expression, resulting in distinct phenotypic changes [21,22]. Moreover, plants actively employ their own sRNAs to suppress the gene expression of a wide range of pathogens [9,23,24]. The current understanding is that the exchange of “RNA-effectors” has evolved into a comprehensive battleground between plants and numerous pathogens. The adversaries in this conflict employ interfering RNAs and effector proteins to disrupt the RNAi apparatus.

Understanding how plants amplify and transfer their own or acquired interfering RNAs to harmful pathogens is not only of fundamental interest [25], but also of applied relevance. Beyond advancing our knowledge of pathogenesis mechanisms, this study lays the groundwork for novel plant defence strategies against pathogenic fungi. These strategies, which were previously focused on viruses and insects, involve host-induced gene silencing (HIGS) and spray-induced gene silencing (SIGS) [25]. HIGS involves the stable expression of a target double-stranded RNA (dsRNA) or high-performance RNA [26,27,28,29] through genetic modification of plants. Conversely, SIGS uses the exogenous delivery of a target molecule produced using bacteria or cell-free transcription systems [30,31,32,33]. While HIGS facilitates a consistent dsRNA supply, eliminating logistical challenges associated with external production, delivery, and stabilisation, SIGS offers greater flexibility. It is worth noting that not all plant species can achieve stable transformants [34]. Furthermore, public concern and legislative restrictions on genetically modified organisms (GMOs) persist in numerous countries [35]. Therefore, choosing definitively between the two strategies is complex, prompting researchers to actively investigate both options. Compared with chemical pesticides, both HIGS and SIGS offer significant advantages, such as target gene specificity and high selectivity, which can reduce the environmental impact [36].

Despite abundant proof-of-concept studies demonstrating the efficacy of SIGS and HIGS against fungi or viruses, these applications seldom progress beyond laboratory settings. In contrast, industrial strategies using similar mechanisms for insect control are actively promoted. A significant limitation is the current lack of knowledge regarding the exchange of RNA between plants and various pathogens at the cellular level. How is dsRNA delivery guaranteed? Is it via extracellular vesicles [37], or could other pathways be involved [38]? What mechanism selects the interfering RNA molecules for transport [39], and where does the processing of long dsRNAs occur [40]? The answers to these questions may vary depending on the host–parasite combination and accurately answering them is crucial for obtaining reliable results.

In this review, we focus primarily on research articles published in international peer-reviewed scientific journals over the last 10 years. Older entries and book chapters are mainly referenced when citing well-established facts or tracing back the history of a specific research topic. References to other review articles are included for their conclusions or to discuss major topics beyond the scope of this review. We aim to examine naturally occurring instances of cross-kingdom RNAi, with induced gene silencing studies incorporated primarily for their utility in showcasing the diversity of RNA movement pathways within plants and microorganisms. Moreover, these studies highlight the practical relevance of this otherwise fundamental topic. We acknowledge that we do not cover all advancements in SIGS and HIGS over the last decade, as that warrants an independent review.

The purpose of this review is to provide a comprehensive analysis and classification of the most up-to-date research on the cross-kingdom transmission of RNA during plant infections caused by a variety of pathogens, including fungi, oomycetes, viruses, viroids, and bacteria. Three key areas are explored: (1) the mechanisms by which plants use interfering RNAs to respond to threats, (2) the utilisation of interfering RNAs by pathogens to optimise host colonisation, and (3) the potential contributions of these findings to the development of innovative plant defence strategies. Each section describes how plants interact with a specific group of pathogens and concludes with a brief summary of future research directions.

## 2. Viruses

Viruses are a primary cause of plant diseases, resulting in significant yield losses. The economic impact of viral diseases is estimated to be approximately $30 billion annually [41,42]. While plant viruses share the obligate intracellular parasitic nature of other viruses, they exhibit unique characteristics due to the distinct structure of plant cells. Most of these organisms exhibit a rod-shaped morphology, lack a lipid envelope, and typically store genetic information in the form of single-stranded RNA (ssRNA) [2,43], although alternative variations are also possible. Plant viruses face a significant barrier regarding transmission between organisms. Unlike animal viruses, which benefit from host mobility for effortless spread, plant viruses must overcome the immobility of their hosts [44]. They typically address the challenge of colonising new plants by using animal vectors, such as insects or nematodes [2,45,46,47]. Once inside, the virus spreads from the primary inoculation site through plasmodesmata [44,48,49]. Given that the virus colonises the entire host, these diseases are considered systemic in nature [50]. Additionally, it should be noted that viral and viroid diseases are chronic, and their potential to be transmitted through vegetative propagation of plants [51,52] makes them especially hazardous [50].

Plants primarily use the RNAi mechanism to defend against viruses [2]. During replication, RNA-containing viruses produce dsRNA intermediates that are specifically recognised by RNAi proteins. The DCL family of proteins, specifically DCL2 and DCL4, plays a significant role in this context. These proteins cleave viral RNA into 22- and 21-nucleotide fragments (siRNAs), which are subsequently utilised by AGO proteins. This phenomenon has been demonstrated in the *Arabidopsis thaliana* model [53]. The contribution of these ribonucleases to viral RNA fragmentation varies, with DCL4 being the main catalyst and DCL2 contributing approximately 20% to siRNA production and operating only in the presence of DCL4 [54,55]. Nevertheless, both enzymes are crucial for plant immunity, and the elimination of either one significantly increases plant susceptibility to viral infections [53].

However, the mere act of cleaving viral RNA into siRNA is insufficient to achieve the protective effect. This suggests that these ribonucleases perform an initiating rather than decisive function in antiviral defence. DCL3 also participates in cleaving viral RNA, producing 24-nucleotide siRNAs. This protein specifically targets transposons and repetitive elements [56]. Unlike DCL2 and DCL4, DCL3 remains active during plant infection by DNA viruses [56]. Substrates also differ among the family: long, perfectly paired RNAs serve as substrates for DCL2, DCL3, and DCL4, whereas DCL1 cleaves short, incompletely paired RNA molecules into 21- and 22-nucleotide fragments [53]. Overall, it is worth noting that despite some substrate differences, all plant DCL proteins participate in defence against RNA and DNA viruses. In both cases, a full spectrum of siRNAs characteristic of the operation of all aforementioned ribonucleases is detected. Although RNAi exists in all eukaryotes, plants possess the highest diversity of DCL proteins. The quantity of ribonuclease is crucial for achieving a specific response to different viral infections [53,56].

Originating from different DCLs, siRNAs interact with AGO proteins to form the RISC. This complex initiates a series of RNAi reactions and/or RNA-directed methylation of viral DNA, ultimately terminating viral particle synthesis. However, viruses have evolved strategies to evade this security system using viral suppressors of RNAi. These suppressors hinder siRNA formation, disrupt AGO and DCL functions, and modify epigenetic patterns of the viral or host genome [57,58,59,60].

Currently, no universally effective techniques exist for controlling viral diseases, especially in the field. Consequently, efforts are taken to cultivate resistant varieties through breeding and genetic engineering techniques [50]. In agriculture, the primary objective is preventing pathogen transmission. This entails promptly identifying and removing diseased plants, disposing of biomass at the end of the growing season, sterilising tools and equipment, and managing insect vectors [61,62]. Progress in applying elicitors to enhance plant immunity has been limited, and adoption rates remain low [63]. Therefore, developing alternative methods to defend plants from viral diseases is exceptionally relevant.

Previously, the sole method for controlling viral infections in plants involved the application of chemical virocides [64]. Although effective in practice, these compounds displayed evident phytotoxic and teratogenic properties [65], impeding their agricultural implementation. However, they remain useful under controlled laboratory conditions for generating healthy plants from infected materials [50].

The introduction of RNAi-based plant defence mechanisms has brought about a shift in the status quo, offering potential strategies for pathogen management via HIGS and SIGS. These methods can also be employed against viroids. In HIGS, the construct is inserted directly into the plant genome. This imposes certain limitations on agricultural use due to regulations regarding GMOs. However, successful implementation of HIGS for controlling cereal viruses has been reported [66]. For instance, Fahim et al. [67] developed wheat resistant to wheat streak mosaic virus (WSMV) by incorporating a miRNA-encoding construct into its genome. This design focused on conserved regions of the WSMV while avoiding non-targeted regions of the wheat genome. Consequently, the plants exhibited no signs of infestation, and their sap did not infect non-transgenic plants that lacked resistance. Similarly, Akbar et al. described success with *Sugarcane mosaic virus* [68]. They incorporated a construct into the rice genome to facilitate the expression of high-performance RNA targeting both the virus envelope protein and proteinase genes. Due to elevated expression of specific 21–24 nucleotide siRNAs, the transgenic plants demonstrated enhanced resistance against cane mosaic virus when compared to wild-type plants.

The augmented safety and prevention of plant genome manipulation have led to the broader application of SIGS in safeguarding plants from viruses [64,69]. Effective measures have been reported for both monocotyledonous and dicotyledonous plants [70,71,72,73]. Although challenges remain regarding stability and delivery methods for exogenous dsRNA, SIGS remains more attractive for field implementation compared to HIGS. This is primarily due to its superior environmental safety and reduced consumer concerns regarding agricultural products [64].

In summary, the plant RNAi machinery offers a viable route for viral control via genome editing or exogenous RNA application. The former provides continuous defence, while the latter is more versatile and less hindered by public concern. This versatility is crucial, as the high mutation rate of viruses allows them to overcome HIGS-mediated resistance in relatively few generations [74]. Combined application of both methodologies is advantageous: HIGS targets the multiple conserved viral protein-coding sequences simultaneously [74], whereas SIGS facilitates rapid responses to emerging threats. Regrettably, current research often focuses solely on a single method.

## 3. Viroids

Viroids are circular RNA plant pathogens, approximately 250 to 400 nucleotides in size, that lack known protein-coding regions and are replicated through a rolling-circle mechanism using host plant enzymes [75]. Viroids are classified into two families, *Pospiviroidae* and *Avsunviroidae*, based on whether they parasitise the plant nucleus or chloroplasts, respectively [76].

During their life cycle, viroids produce dsRNA intermediates. Additionally, their genomic RNA contains a substantial number of hairpins. Thus, they can be recognised by plant DCL proteins and serve as a source of viroid-derived small RNAs (vd-sRNAs) [22]. Infection development and symptom manifestation exhibit a twofold feature. On the one hand, infection enables the plant to activate the RNAi apparatus against the viroid. On the other hand, it exposes its own genes to vd-sRNA-mediated silencing. Katsarou et al. [77] demonstrated the first variation in this interaction. In their study, the disruption of genes encoding the DCL2 and DCL3 proteins in *Nicotiana benthamiana* increased the viroid titre in plants infected with potato spindle tuber viroid (PSTVD). Conversely, the knockout of *dcl4* resulted in a decline in the viroid load [77]. In parallel, Suzuki et al. [78] demonstrated in tomatoes that the simultaneous inhibition of gene expression for DCL2 and DCL4 resulted in the conversion of a PSTVD-resistant cultivar into a susceptible one, ultimately causing plant mortality [78]. The findings reveal the crucial contribution of specific DCL proteins to viroid RNA fragmentation, thereby exerting a substantial influence on plant control of PSTVD. However, plants within the same family can employ varying combinations of DCL endonucleases to counteract PSTVD. For instance, in *N. benthamiana*, DCL4 likely exhibits inefficient viroid processing while competing with the dominant DCL2 and DCL3. In contrast, the absence of DCL4 in tomatoes diminishes plant defence.

**Figure 2:**
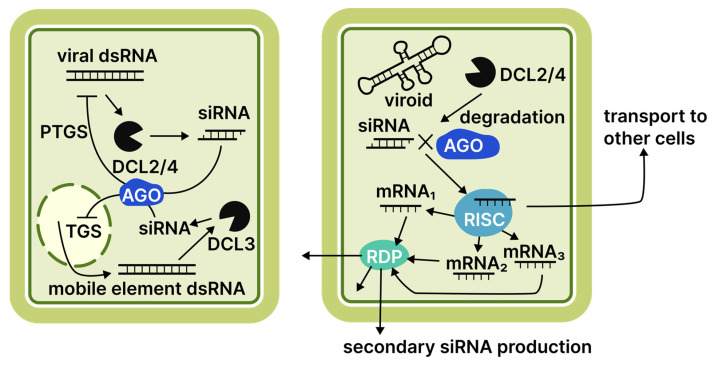
Comparison of virus- and viroid-induced RNAi responses in plants. Numbers adjacent to protein family names indicate specific proteins discussed in the literature reviewed. Abbreviations: DCL, Dicer-like protein; AGO, Argonaute protein; RDR, RNA-dependent RNA polymerase; RISC, RNA-induced silencing complex; TGS, transcriptional gene silencing; PTGS, post-transcriptional gene silencing.

Another aspect of viroid participation in host RNAi pathways is the partial complementarity between vd-sRNAs and cellular mRNAs. This partial complementarity can be sufficient to impede translation, resulting in specific phenotypes [22,79,80]. This mechanism is highly significant for viroid virulence. Even small nucleotide substitutions disrupting complementarity can profoundly affect the severity of characteristic symptoms [21]. Beyond the direct involvement of vd-sRNAs in RISC, the induction of post-transcriptional gene silencing (PTGS) can trigger the generation of phased siRNAs on the mRNA substrate through the recruitment of RNA-dependent RNA polymerase 6 (RdR6) [81]. This process could potentially result in a more efficient expression suppression and the systemic spread of phased siRNAs within the phloem. However, validation of this effect is based solely on indirect evidence, such as identifying several phased siRNAs linked to PSTVD infection and sequencing data showing viroid-derived siRNAs capable of suppressing host genes [81]. Conversely, some evidence suggests RdR6 is necessary to reduce viroid virulence [82]. Unlike plant antiviral defence, the RNAi machinery may be a liability against viroids, serving not only as an anti-viroid tool but also as a plant-damaging agent. The role of the RNAi machinery in the plant response against viruses and viroids is summarised in Figure 2.

Another noteworthy characteristic of viroids, specifically PSTVD, is their ability to spread when potatoes are infected with the oomycete *Phytophthora infestans*. Afanasenko et al. [83] reported that PSTVD can invade the *P. infestans* mycelium during the inoculation of viroid-infected potatoes and persist in the oomycete culture for several passages [83]. Another study established that viroid transmission to oomycetes occurs under natural field conditions [84]. Unfortunately, a lack of data hinders our understanding of viroid replication mechanisms in this atypical host and its influence on the RNAi pathways of *P. infestans*.

Another study provides a detailed account of utilising SIGS and HIGS for viroid management [85]. In summary, despite some achievements in enhancing plant resistance to PSTVD through HIGS, this method does not provide a complete cure and applies to a limited range of plants [85]. Moreover, SIGS is ineffective in viroid control, necessitating continuous and costly treatment. Therefore, effective viroid control requires measures to halt propagation and contagion [86]. Since viroids do not encode proteins and form quasispecies populations with varied nucleotide sequences in a single plant [87], RNAi tools should target the transcription rate of the plant’s own genes rather than suppress them [88]. Further studies of the infested plant transcriptome [81] are required to identify genes responsible for symptoms. These genes could then be silenced or their mRNA sequences altered to prevent interaction with the viroid-derived siRNA.

## 4. Bacteria

Agricultural sectors suffer considerable economic losses from plant bacterial diseases. Unlike other microorganisms, pathogenic bacteria are sporadic in occurrence. However, a severe infestation can result in catastrophic consequences, potentially leading to a yield loss of up to 100% [89]. While global data on the economic damage from bacteriosis are unavailable, yield losses in the USA alone are estimated at tens of billions of dollars [90]. It is often challenging to establish the root cause of a disease in the field, primarily due to the active involvement of secondary pathogens, which impedes the assessment of the causative agent. Nevertheless, researchers have effectively ranked the most detrimental pathogenic bacteria for plants. The top five include *Pseudomonas syringae* pathovars, *Ralstonia solanacearum*, *Agrobacterium tumefaciens*, *Xanthomonas oryzae* pv. *oryzae*, and *Xanthomonas campestris* pathovars [91]. Naturally, many more economically significant pathogens warrant consideration.

The primary mechanisms for bacterial transmission are wind and rain [92]. However, humans also play a crucial role in agricultural settings. Activities such as pruning, grafting, and the use of non-sterile tools contribute to pathogen transmission from plant to plant [93]. Animals, particularly insects, ticks, and birds, can also act as carriers of bacterial diseases [90]. Similarly to other pathogens, the primary approach to managing plant bacterioses involves preventive measures, such as tool sterilisation and inoculum control [90]. When signs of bacterial diseases are detected in the field, the application of chemical treatments is typically similar to shutting the stable door after the horse has bolted. While copper-based compounds may offer some benefits, their efficacy is generally regarded as suboptimal [94,95]. A critical issue is the increasing problem of bacterial resistance, which affects both copper-containing formulations and commonly used antibiotics [90,96]. This necessitates exploring alternative means of plant protection.

**Figure 3:**
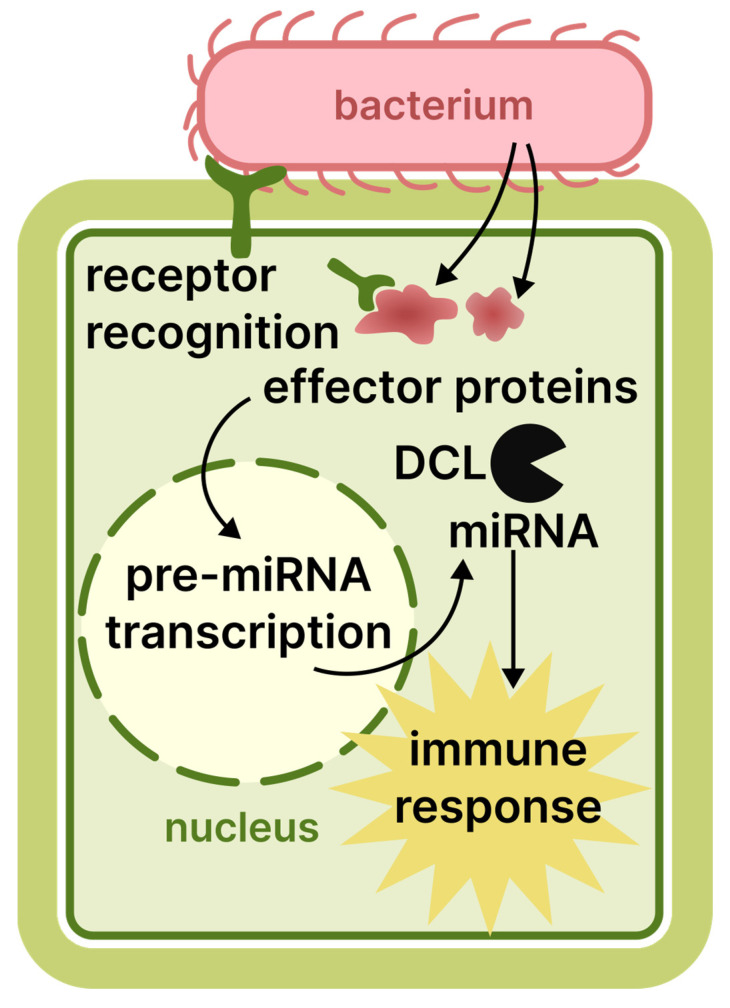
The role of RNAi in the plant immune response to bacterial infection.

Plants have defence mechanisms against bacteria that involve RNAi, similar to their responses to other pathogens. However, RNAi is not their primary defence method, and differences exist in this mechanism compared to the response against viruses and viroids. Infection begins when the bacterium enters the plant, typically through natural pathways such as stomata or, most commonly, by taking advantage of physical damage. Bacterial pathogen-associated molecular patterns are the main stimulus for the plant response. However, pathogens have developed mechanisms to overcome this step. They introduce diverse effector molecules into plant cells, subsequently modifying the transcriptome and proteome of the host cell. This alteration increases the susceptibility of the plant to infection [2]. Upon detecting the effectors, the plant swiftly activates its defence mechanisms, which involve the production of various endogenous siRNAs and miRNAs. These molecules are responsible for finely adjusting and directing the plant’s immune response towards the pathogen [97,98]. A study on *A. thaliana* revealed that plants with mutations in the RNAi genes *dcl* and *hen* exhibited increased bacterial growth when infected with non-virulent forms of *P. syringae*. Furthermore, a shortage of immune-response-related miRNAs was reported in mutant variations [99]. Additionally, the same gene mutations can cause an observed increase in resistance to other pathogens, such as *A. tumefaciens* [100]. Moreover, bacterial effectors can suppress the plant RNAi system by binding to DCL or AGO proteins [101]. Therefore, while the plant RNAi machinery may not directly target bacterial genes, it still plays a significant part in the complex immune response against bacterial infection. This principle is summarised in Figure 3.

In summary, research into the plant immune response to bacterial infection using RNAi is limited, with few advances in immune enhancement via SIGS or HIGS. The reason is that bacteria lack their own RNAi machinery, which limits their ability to interact with the host plant via small RNAs. It is unlikely that a specific RNAi tool will be developed to deal permanently with a particular pathogenic bacterium. More likely, the outcome will be a moderate overall enhancement of antibacterial resistance. From this perspective, more readily applied chemicals that act as immune inducers are receiving considerable attention [102,103,104], with RNAi utilisation confined to fundamental research for now.

## 5. Fungi

The realm of true fungi contains an extensive assortment of organisms, including numerous economically vital plant pathogens like *B. cinerea*, *Fusarium graminearum*, *Puccinia graminis*, and many more. Despite apparent similarities, significant biological and biochemical differences exist, particularly in the RNAi mechanism [40]. These differences contribute to the ineffectiveness of a one-size-fits-all approach to fungal control through HIGS or SIGS methods [105]. Instead, the methods must be adapted to each host/pathogen pathosystem separately, which presents considerable obstacles.

More than a decade has passed since the first identification of cross-kingdom exchange of interfering RNAs between *B. cinerea*, *A. thaliana*, and tomato (*Solanum lycopersicum*) [17]. Nevertheless, except for genetically modified plants producing artificial dsRNAs, there are relatively few definitively confirmed cases of interfering RNA transfer between plants and fungi [9,18,23,106]. In contrast, abundant evidence demonstrates the effectiveness of HIGS. This approach uses constructs aimed at suppressing pathogen gene expression, resulting in a substantial decrease in transcription levels and virulence [26,27,28,29,107,108,109].

Why is there such a significant preference for artificial HIGS in the literature? Establishing a relationship between the increased copy number of a specific sRNA in one organism and the reduced copy number of a potentially targeted mRNA in another organism during infection is laborious. Evidence supporting this relationship often requires genetic modification of both the host plant and the fungi (Table 1) [9,18,23,106]. For instance, Weiberg et al. [17] successfully obtained a *B. cinerea* line with simultaneous knockouts of the *dcl1* and *dcl2* genes. The necessity for such manipulations restricts the pool of host/pathogen pathosystems that can be studied, as established protocols are not available for all plants and pathogens of interest. Furthermore, the findings may have limited replicability. As a case in point, the study conducted by Qin et al. [110] revealed that the *B. cinerea* double mutants with mutations in both the *dcl1* and *dcl2* genes did not exhibit any abnormal phenotype and showed reduced virulence, in contrast to the results reported previously [17,106]. Based on the analysis of previous data and findings from experiments involving the deletion of fungal genome regions from which sRNA precursor molecules are transcribed, Qin et al. [110] concluded that cross-kingdom exchange is insignificant in *B. cinerea* pathogenesis.

It is challenging for an external researcher attempting to elucidate the mechanisms of cross-kingdom RNA exchange and its relevant proteins to reconcile these conflicting data. However, the significance of *dcl1* and *dcl2* genes in pathogenesis has been confirmed both in organisms related to *B. cinerea* [111] and in other necrotrophic fungi [112]. In addition to contradicting earlier studies, the research by Qin et al. [110] also demonstrates that a simple correlation between an increase in the copy number of a given sRNA and a simultaneous decrease in the copy number of its potential target mRNA is insufficient to prove that silencing is occurring. New research on the AGO proteins of the fungus and their activity against plant defences could resolve the existing confusion regarding the importance of RNAi during *B. cinerea* infection [113]. On the one hand, they are required for the accumulation of fungal cross-kingdom small RNAs. On the other hand, they provide a platform for plant miRNAs to silence the fungal genes. Their dual role as both a liability and a virulence mechanism explains why their knockout does not appear to affect the virulence of the fungus. Further research is required to investigate whether this ambiguous effect occurs in hosts other than *Arabidopsis* and tomato, and whether it is cultivar- or strain-specific.

**Table 1:** Examples of cross-kingdom RNAi against fungi.

Host Plant	Pathogen	Target Genes	Application Method	Reference
*Triticum aestivum* (wheat)	*Fusarium asiaticum*	*myo5*	SIGS	[39]
	*Puccinia striiformis*	*pr2*	HIGS	[18]
*Malus hupehensis*	*Botryosphaeria dothidea*	*miR159a*	HIGS	[9]
*Gossypium hirsutum* (cotton)	*Verticillium dahliae*	*clp-1* and *hiC-15*	natural plant microRNAs	[23]
*Zea mays* (maize)	*Aspergillus flavus*	*p2c*	HIGS	[26]
*Oryza sativa* (rice)	*Rhizoctonia solani*	*AGLIP1*	HIGS	[27]
	*Magnaporthe oryzae*	*RGS1*, *MgAPT2* and *LHS1*	HIGS	[29]
*Hordeum vulgare* (barley)	*Fusarium species*	*CYP51*	HIGS	[105]
*Musa* spp. (banana)	*Fusarium oxysporum*	*velvet* genes and *FTF1*	HIGS	[106]
*Solanum tuberosum* (potato)	*Phytophthora infestans*	*Avr3a*	HIGS	[112]

When it comes to artificial HIGS and, particularly, SIGS, the primary obstacle is not proving silencing, as quantitative RT-PCR and assessment of infection severity can suffice [114,115], but choosing the appropriate target genes. In the most straightforward scenario, this would pertain to a recognised and annotated gene, for instance, one responsible for toxin synthesis [26]. In certain cases, feasibility tests on the suppression of the target gene in fungal knockout lines are conducted [28]. However, the most common approach is to produce a substantial quantity of genetically modified plants or preparations of synthetic inducible gene systems for SIGS targeting different predicted fungal genes [27]. This approach does not require genetic modification of the pathogen and remains efficacious even where the genome and transcriptome are insufficiently annotated.

In this context, it is noteworthy that the effector genes of fungi exhibit a greater variation and poorer annotation than conserved sequences. Nonetheless, these genes stand out as highly promising targets for SIGS, primarily due to the reduced probability of off-target effects caused by their complementary dsRNAs [34]. Utilising spray-induced suppression methods to inhibit the expression of conserved genes, such as *chitin synthase 3a* and *dcl1/2*, is a commendable proof-of-concept for innovative treatment strategies [111]. Nevertheless, the implementation of such formulations in field conditions may pose unpredictable hazards [34]. HIGS displays heightened resistance to target restrictions, given the low levels of dsRNA or sRNA released into the external environment. However, it should be noted that HIGS plants are considered GMOs and thus are subject to the limitations associated with this classification [116].

While research on fungal-to-plant RNA transfer is of significant interest, studies specifically investigating the mechanisms of plant-to-fungal interfering RNA transfer can contribute to the more efficient development of HIGS and SIGS. The first published study [23] elucidated the function of cotton miRNA159 and miRNA166 in suppressing the expression of two genes in the parasitic fungus *Verticillium dahliae*. Of particular significance was the observation that modifying the landing site of a specific miRNA led to increased pathogen virulence and target gene expression, thereby impeding silencing. Recently, the role of a miRNA, Os-miR169y, in the non-host defence of rice against *Sclerotinia sclerocium* was reported [117]. It was found to be overexpressed during the *S. sclerocium* attempt to infect rice plants, and its silencing promoted the infection. Moreover, exogenous application of this miRNA on the rapeseed or *A. thaliana* plants, as well as its expression in *A. thaliana*, enhanced their defence against the fungus. This research demonstrates that the small RNAs involved in non-host resistance may indicate potential SIGS or HIGS targets for protecting host plants.

The transmission pathways were not examined in the initial studies. Nevertheless, subsequent research [37] provided a comprehensive characterisation of TET8 and TET9 tetraspanins. These proteins were found to play a crucial role in the formation of sRNA-transporting extracellular vesicles (EVs) in *A. thaliana*. Furthermore, the EVs were secreted and subsequently taken up by *B. cinerea*. Not only do EVs contain envelope proteins, but they also harbour substantial amounts of RNA-binding proteins, such as AGO1. The selective binding of export-targeted RNAs by those proteins potentially suggests their role in small RNA sorting [39]. A recent study on the apple *Malus hupehensis* [9] demonstrated the capacity of plant miRNAs to encapsulate into EVs and infiltrate the pathogenic fungus. Only one of the two miRNAs, miRNA159a, which was abundantly present in EVs, was found to affect pathogen virulence.

Apart from the secretion process involving vesicles, scientific discourse exists on an alternative, vesicle-independent pathway for delivering RNA from plants to pathogens. A study by Zand Karimi et al. demonstrated the existence of small RNAs and long non-coding RNAs in the apoplastic fluid of *A. thaliana* [38]. The authors placed significant emphasis on the widespread presence of N6-methyladenine-mediated post-transcriptional modification, specifically in extracellular RNAs. They suggested this modification could be a marker for secretion that does not rely on EVs. How crucial is this secretion for cross-kingdom RNA metabolism? When considering barley *Hordeum vulgare*, specifically in the context of SIGS, several publications have demonstrated that dsRNAs travel through the plant and enter *F. graminearum*, leading to a significant decrease in virulence [118]. Moreover, the EVs of barley were found to contain negligible amounts of exogenous dsRNAs and their derivatives [11], and applying them did not affect the treatment of the fungus [10]. The authors highlighted the potential for an interesting comparison between RNA representation in HIGS and SIGS vesicles from plants of the same species [10]. However, research on this specific element is limited.

Therefore, cross-kingdom RNA exchange between plants and fungi can occur via either the secretion into EVs or without their involvement. The precise mechanism of RNA selection for vesicle packaging remains incompletely elucidated. Nonetheless, it should be emphasised that even in the case of RNAs expressed in the transgenic *A. thaliana* plant, over 70% of HIGS-derived siRNAs are found outside the vesicles [11]. It can be concluded that in planta dsRNA transcription is not the exclusive determinant for packaging. Another point regarding RNA delivery mechanisms using SIGS is the entry of RNA directly into the pathogen from the environment. Despite not being related to cross-kingdom exchange, this mechanism merits attention [105].

This work ultimately stresses the necessity of meticulously adjusting RNAi technologies to each pathosystem. Additional research should focus not only on identifying novel target genes and methods for cross-kingdom RNA delivery, but also on testing established methods on diverse cultivars, strains, and closely related species. This is laborious work with little prospect of breakthrough discoveries, but without it, antifungal RNAi is unlikely to acquire significant practical implications. Works like [105] have the potential to integrate the currently fragmented understanding of fungal-plant RNA interactions. However, they should be carefully designed to avoid further discrepancies. The simultaneous application of dsRNA and the pathogen inoculum, as discussed in the article, may be the source of such confusion [40], as it eliminates the possibility of RNA translocation throughout the plant. This may explain why the results of Qiao et al. regarding the *P. infestans* susceptibility to spray-induced gene silencing conflict with those of Sundaresha et al. [115], who maintained a 24 h interval between dsRNA spraying and the inoculation.

## 6. Oomycetes

**Figure 4:**
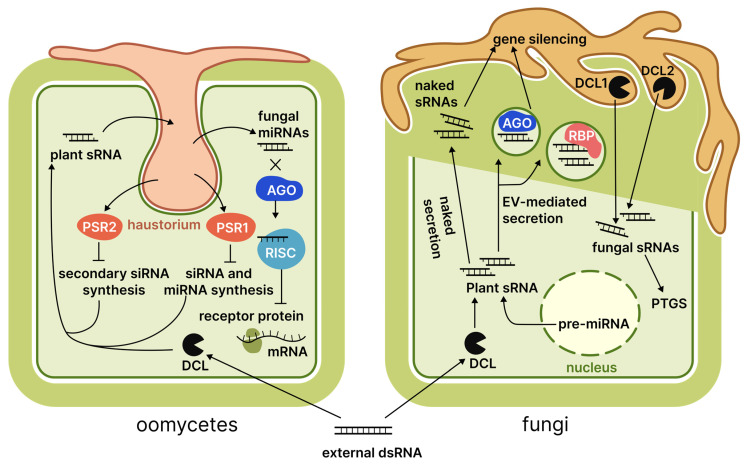
Plant RNA interactions with fungi and oomycetes. Numbers adjacent to protein family names indicate specific proteins discussed in the literature reviewed. Abbreviations: DCL, Dicer-like protein; AGO, Argonaute protein; RISC, RNA-induced silencing complex; PSR, *Phytophthora* suppressor of RNA silencing; RBP, RNA-binding protein; PTGS, post-transcriptional gene silencing; EV, extracellular vesicles.

The oomycetes, belonging to the Stramenopiles clade, include fungus-like organisms such as *Phytophthora* and *Peronospora*. These organisms differ from true fungi in their origin, structure, and biochemical processes, notably in possessing cellulose-based cell walls rather than chitin. Numerous oomycetes act as obligate parasites of agricultural plants, resulting in substantial economic losses [119]. While the principles of SIGS and HIGS apply to fungi, oomycetes employ a broader range of RNAi mechanisms during pathogenesis, necessitating a dedicated section. This observation is particularly relevant for RNAi suppressor proteins, including PSR1 [120] and PSR2 [24], which have been detected in *P. infestans*. Qiao et al. [120] explained the influence of PSR1 on miRNA and siRNA synthesis in *A. thaliana* through its interaction with a plant protein named PSR1-interacting protein. Furthermore, the detrimental effects of PSR1 were observed in transgenic plants even in the absence of infection, leading to stunted growth, delayed flowering, and reduced seed production. In contrast, PSR2 functions to directly suppress the generation of secondary siRNAs, thereby preventing their inter-kingdom transmission from *A. thaliana* to *P. infestans* [24]. During the biotrophic phase of infection, an increase in the expression of the gene coding for the PSR2 protein was observed, which suggests its importance in counteracting the host plant defence systems [121]. The presence of these proteins signifies the relevance of inter-kingdom RNA exchange in oomycete pathogenesis. Like fungi, oomycetes are seemingly involved in an evolutionary arms race against their hosts on the RNAi field. Key findings regarding these confrontations are summarised in Figure 4.

Besides the impacts effector proteins have on host RNAi pathways, oomycetes employ cross-kingdom RNA exchange to hinder their defences. For example, *P. infestans* counteracts the membrane receptor StABH1 in potatoes by suppressing the expression of target genes using miR8788 [19]. Similarly, *Hyaloperonospora arabidopsidis* employs a minimum of three sRNAs that are co-precipitated with host AGO1 proteins through immunoprecipitation and demonstrate complementarity to *A. thaliana* defence genes [122]. An interesting approach was implemented to tackle cross-kingdom RNA exchange [122]: short-tandem-target-mimic (STTM) RNA complementary to *Hyaloperonospora arabidopsidis* small RNAs was expressed in the plant and used to bind them, resulting in reduced virulence.

Another notable characteristic of oomycetes is their active utilisation of sRNAs, which possess an atypical length of 25–26 nt, to silence their effectors. Intriguingly, siRNA can repress the expression of up to 31% of the relevant genes [123]. This mechanism may have evolved to eliminate proteins identified by the host plant, but it has expanded, which could decrease oomycete virulence [122]. As demonstrated by [122], disrupting a central pathogenesis mechanism can lead to unanticipated and undesirable consequences.

In summary, regarding oomycetes, it may be more productive to concentrate on eliminating the host plant susceptibility factors [123], rather than pursuing further studies on suppressing their pathogenicity. This approach should allow researchers to pay less attention to the reported contradictions regarding the limited uptake of environmental RNA by oomycetes [105] or their abnormal RNAi mechanisms [122]. Therefore, it is essential to investigate cross-kingdom RNAi between plants and oomycetes further, as it may offer new insights into mechanisms of plant susceptibility.

## 7. Conclusions

Plant infection by viruses, viroids, fungi, and oomycetes involves a complex exchange of small RNAs, with RNAi playing a pivotal role in determining the disease outcome. Given that plant immune responses are primarily centred on RNAi reactions, the ability of pathogens to overcome these defence mechanisms through anti-silencing mechanisms is critical for successful colonisation. The most significant manifestation of this system can be observed in the interaction between plants and viruses or viroids, given the consistent presence of dsRNA, a primary target of RNAi. In the battle against fungi and oomycetes, both sides actively employ gene silencing and exchange small RNA to combat each other, and the effectiveness of these strategies could determine the outcome of the confrontation. Ongoing advancements in molecular biology techniques have facilitated investigations into the mechanisms of plant resistance to pathogens. Additionally, this knowledge can be applied in practical areas, such as identifying innovative strategies to safeguard crops from disease.

It should be noted that, even though most innovations are not yet suitable for practical applications, they show promising potential for scaling up. This becomes even more crucial due to the issue of pathogens becoming resistant to various chemicals used as the main line of defence in plants. Another major advantage of the suggested RNAi-based approaches is their remarkable specificity and favourable biosafety profile regarding the environment and the end user, particularly in the context of agricultural products. Further research is clearly warranted to explore RNA transfer in plant-pathogen interactions, with a specific focus on the role of RNAi in bacterial defence. Advancing this field will facilitate integrated approaches for agricultural protection, leading to reduced crop loss and enhanced food security in the face of a growing global population.

## Data Availability

No new data were created or analyzed in this study. Data sharing is not applicable to this article.
